# Di-μ-sulfato-bis­{[bis­(3,5-dimethyl­pyrazol-1-yl)methane]copper(II)}

**DOI:** 10.1107/S1600536808027840

**Published:** 2008-09-06

**Authors:** YanLi Wu, XingCong Wang, XiuLi You

**Affiliations:** a Jiangxi Key Laboratory of Organic Chemistry, Jiangxi Science and Technology Normal University, Nanchang 330013, People’s Republic of China

## Abstract

The mol­ecule of the title compound, [Cu_2_(SO_4_)_2_(C_11_H_16_N_4_)_2_], sits on a center of symmetry. The Cu^II^ atom has a distorted trigonal–bipyramidal coordination geometry comprising three O atoms of the two symmetry-related SO_4_^2−^ anions and two N atoms from one bis­(3,5-dimethyl­pyrazol-1-yl)methane ligand.

## Related literature

For related literature, see: Arnold *et al.* (2001 ▶); Dhar *et al.* (2004 ▶); Endres *et al.* (1984 ▶); Hatzidimitriou *et al.* (2006 ▶); He & Han (2006 ▶); Springsteen *et al.* (2006 ▶); Tamasi & Cini (2003 ▶); Thompson *et al.* (1998 ▶).
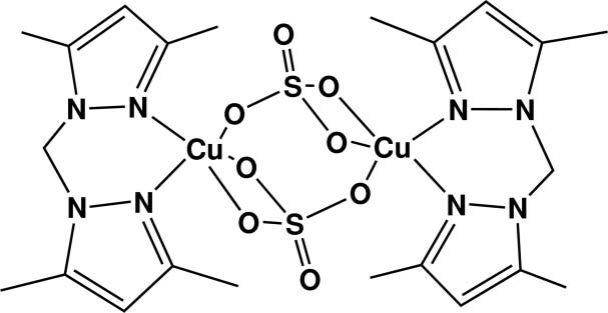


## Experimental

### 

#### Crystal data

[Cu_2_(SO_4_)_2_(C_11_H_16_N_4_)_2_]
*M* *_r_* = 727.76
Monoclinic, 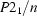

*a* = 7.5293 (15) Å
*b* = 10.734 (2) Å
*c* = 17.740 (4) Å
β = 99.73 (3)°
*V* = 1413.2 (5) Å^3^
*Z* = 2
Mo *K*α radiation
μ = 1.71 mm^−1^
*T* = 291 (2) K
0.22 × 0.19 × 0.19 mm

#### Data collection

Rigaku Mercury diffractometer
Absorption correction: multi-scan (Jacobson, 1998 ▶) *T*_min_ = 0.704, *T*_max_ = 0.737
13344 measured reflections
2580 independent reflections
2253 reflections with *I* > 2σ(*I*)
*R*_int_ = 0.039

#### Refinement

*R*[*F* ^2^ > 2σ(*F* ^2^)] = 0.045
*wR*(*F* ^2^) = 0.112
*S* = 1.07
2580 reflections
194 parameters
H-atom parameters constrained
Δρ_max_ = 0.46 e Å^−3^
Δρ_min_ = −0.42 e Å^−3^

### 

Data collection: *CrystalClear* (Rigaku/MSC, 2001 ▶); cell refinement: *CrystalClear*; data reduction: *CrystalStructure* (Rigaku/MSC, 2004 ▶); program(s) used to solve structure: *SHELXS97* (Sheldrick, 2008 ▶); program(s) used to refine structure: *SHELXL97* (Sheldrick, 2008 ▶); molecular graphics: *SHELXTL* (Sheldrick, 2008 ▶); software used to prepare material for publication: *SHELXTL*.

## Supplementary Material

Crystal structure: contains datablocks I, global. DOI: 10.1107/S1600536808027840/cs2085sup1.cif

Structure factors: contains datablocks I. DOI: 10.1107/S1600536808027840/cs2085Isup2.hkl

Additional supplementary materials:  crystallographic information; 3D view; checkCIF report

## Figures and Tables

**Fig. 1.**
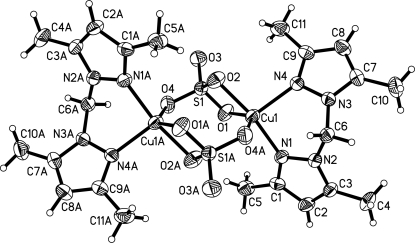
The molecular structure with displacement ellipsoids drawn at the 50% probability level. Atoms labeled with the suffix A are related by the (-x, 1 - y, - z) symmetry operator.

## supplementary crystallographic information

### Comment

SO_4_^2-^ anion-bridged dimeric complexes of Cu(II) are reported extensively (Tamasi & Cini, 2003). In most of these structures the SO_4_^2-^ anion acts as a bidentate bridge (Springsteen *et al.*, 2006; He & Han, 2006; Arnold *et al.*, 2001; Thompson *et al.*, 1998; Endres *et al.*, 1984). However, there are only two known examples of the tridentate bridge form (Hatzidimitriou *et al.*, 2006; Dhar *et al.*, 2004). The crystal structure of the title compound, [Cu(*bdmpm*)(SO_4_)]_2_ (*bdmpm* = bis(1,1-bis(3,5-dimethylpyrazol-1-yl)methane), shows a perfect centrosymmetric dimer, as two {Cu(*bdmpm*)}^2+^ units are bridged by two sulfate anions in the complex (Fig. 1). The Cu···Cu distance is 3.769 (11) Å and the copper atom has a trigonal bipyramidal CuN_2_O_3_ coordination geometry with the sulfate O(2) atom and the N(1) atom as axial ligand atoms.

### Experimental

The reaction of CuSO_4_.5H_2_O (25 mg, 0.1 mmol) with *bdmpm* (22 mg, 0.11 mmol) in MeOH (10 ml) was carried out at ambient temperature for 10 minutes, the mixture was filtered and the filtrate was then left for crystallization.

### Refinement

All H atoms were positioned geometrically and refined using a riding model, with C—H = 0.93–0.97 Å and *U*_iso_(H) = 1.2 times *U*_eq_(C).

### Crystal data

**Table:**

[Cu_2_(SO_4_)_2_(C_11_H_16_N_4_)_2_]	*F*(000) = 748
*M**_r_* = 727.76	*D*_x_ = 1.710 Mg m^−^^3^
Monoclinic, *P*2_1_/*n*	Mo *K*α radiation, λ = 0.71073 Å
Hall symbol: -P 2yn	Cell parameters from 4573 reflections
*a* = 7.5293 (15) Å	θ = 3.0–25.4°
*b* = 10.734 (2) Å	µ = 1.71 mm^−^^1^
*c* = 17.740 (4) Å	*T* = 291 K
β = 99.73 (3)°	Block, green
*V* = 1413.2 (5) Å^3^	0.22 × 0.19 × 0.19 mm
*Z* = 2	

### Data collection

**Table:**

Rigaku Mercury diffractometer	2580 independent reflections
Radiation source: fine-focus sealed tube	2253 reflections with *I* > 2σ(*I*)
graphite	*R*_int_ = 0.039
Detector resolution: 14.6306 pixels mm^-1^	θ_max_ = 25.4°, θ_min_ = 3.0°
ω scans	*h* = −9→9
Absorption correction: multi-scan (Jacobson, 1998)	*k* = −12→12
*T*_min_ = 0.704, *T*_max_ = 0.737	*l* = −21→21
13344 measured reflections	

### Refinement

**Table:**

Refinement on *F*^2^	Primary atom site location: structure-invariant direct methods
Least-squares matrix: full	Secondary atom site location: difference Fourier map
*R*[*F*^2^ > 2σ(*F*^2^)] = 0.045	Hydrogen site location: inferred from neighbouring sites
*wR*(*F*^2^) = 0.112	H-atom parameters constrained
*S* = 1.07	*w* = 1/[σ^2^(*F*_o_^2^) + (0.054*P*)^2^ + 2.0475*P*] where *P* = (*F*_o_^2^ + 2*F*_c_^2^)/3
2580 reflections	(Δ/σ)_max_ < 0.001
194 parameters	Δρ_max_ = 0.46 e Å^−^^3^
0 restraints	Δρ_min_ = −0.42 e Å^−^^3^

### Fractional atomic coordinates and isotropic or equivalent isotropic displacement parameters (Å^2^)

**Table:**

	*x*	*y*	*z*	*U*_iso_*/*U*_eq_	
Cu1	−0.00166 (6)	0.33244 (4)	0.03099 (2)	0.03196 (18)	
S1	−0.08737 (12)	0.41689 (8)	−0.10738 (5)	0.0301 (2)	
O1	−0.2040 (4)	0.3437 (3)	−0.06474 (16)	0.0422 (7)	
O2	0.0920 (3)	0.4113 (3)	−0.05419 (15)	0.0431 (7)	
O3	−0.0756 (4)	0.3645 (3)	−0.18102 (15)	0.0461 (7)	
O4	−0.1457 (4)	0.5477 (3)	−0.11429 (16)	0.0444 (7)	
N1	−0.1409 (4)	0.2717 (3)	0.10764 (17)	0.0316 (7)	
N2	−0.0581 (4)	0.1991 (3)	0.16697 (17)	0.0330 (7)	
N3	0.1848 (4)	0.1141 (3)	0.11191 (17)	0.0316 (7)	
N4	0.1593 (4)	0.1722 (3)	0.04242 (17)	0.0315 (7)	
C1	−0.3116 (5)	0.2839 (4)	0.1176 (2)	0.0343 (9)	
C2	−0.3360 (6)	0.2205 (4)	0.1837 (2)	0.0411 (10)	
H2	−0.4423	0.2152	0.2036	0.049*	
C3	−0.1745 (6)	0.1674 (4)	0.2137 (2)	0.0365 (9)	
C4	−0.1213 (7)	0.0889 (5)	0.2836 (3)	0.0557 (13)	
H4A	−0.0814	0.0089	0.2691	0.084*	
H4B	−0.2230	0.0785	0.3092	0.084*	
H4C	−0.0253	0.1291	0.3175	0.084*	
C5	−0.4425 (5)	0.3586 (4)	0.0638 (2)	0.0433 (10)	
H5A	−0.3832	0.4307	0.0478	0.065*	
H5B	−0.5399	0.3843	0.0888	0.065*	
H5C	−0.4889	0.3089	0.0198	0.065*	
C6	0.1344 (5)	0.1810 (4)	0.1760 (2)	0.0331 (8)	
H6A	0.1751	0.1348	0.2228	0.040*	
H6B	0.1939	0.2615	0.1807	0.040*	
C7	0.2597 (5)	−0.0001 (4)	0.1088 (2)	0.0360 (9)	
C8	0.2818 (6)	−0.0156 (4)	0.0345 (2)	0.0418 (10)	
H8	0.3300	−0.0851	0.0140	0.050*	
C9	0.2187 (5)	0.0921 (4)	−0.0045 (2)	0.0340 (9)	
C10	0.3072 (7)	−0.0825 (4)	0.1768 (3)	0.0509 (11)	
H10A	0.4025	−0.0449	0.2123	0.076*	
H10B	0.3462	−0.1619	0.1609	0.076*	
H10C	0.2034	−0.0936	0.2009	0.076*	
C11	0.2141 (6)	0.1216 (5)	−0.0874 (2)	0.0495 (11)	
H11A	0.0912	0.1305	−0.1123	0.074*	
H11B	0.2701	0.0552	−0.1111	0.074*	
H11C	0.2779	0.1979	−0.0920	0.074*	

### Atomic displacement parameters (Å^2^)

**Table:**

	*U*^11^	*U*^22^	*U*^33^	*U*^12^	*U*^13^	*U*^23^
Cu1	0.0298 (3)	0.0404 (3)	0.0263 (3)	0.00246 (19)	0.0063 (2)	0.00527 (19)
S1	0.0345 (5)	0.0300 (5)	0.0251 (5)	−0.0015 (4)	0.0034 (4)	−0.0014 (4)
O1	0.0415 (16)	0.0482 (17)	0.0366 (15)	−0.0127 (13)	0.0059 (13)	0.0060 (13)
O2	0.0328 (15)	0.064 (2)	0.0313 (14)	−0.0037 (13)	0.0025 (12)	0.0067 (14)
O3	0.063 (2)	0.0462 (17)	0.0285 (14)	0.0050 (14)	0.0053 (14)	−0.0078 (13)
O4	0.0621 (19)	0.0333 (15)	0.0372 (15)	0.0054 (14)	0.0068 (14)	0.0024 (12)
N1	0.0287 (16)	0.0381 (18)	0.0286 (16)	0.0030 (14)	0.0066 (13)	0.0039 (14)
N2	0.0355 (18)	0.0353 (17)	0.0287 (16)	0.0059 (14)	0.0069 (14)	0.0051 (14)
N3	0.0357 (17)	0.0299 (16)	0.0297 (16)	0.0044 (14)	0.0068 (14)	−0.0006 (14)
N4	0.0336 (17)	0.0320 (17)	0.0293 (16)	0.0045 (13)	0.0062 (14)	0.0010 (13)
C1	0.033 (2)	0.034 (2)	0.038 (2)	0.0010 (16)	0.0112 (17)	−0.0067 (17)
C2	0.041 (2)	0.044 (2)	0.043 (2)	−0.0049 (19)	0.0210 (19)	0.0026 (19)
C3	0.044 (2)	0.034 (2)	0.035 (2)	−0.0025 (17)	0.0155 (19)	0.0045 (17)
C4	0.068 (3)	0.059 (3)	0.044 (3)	0.004 (2)	0.023 (2)	0.023 (2)
C5	0.028 (2)	0.057 (3)	0.045 (2)	0.0065 (19)	0.0064 (19)	0.002 (2)
C6	0.039 (2)	0.035 (2)	0.0249 (18)	0.0016 (17)	0.0035 (16)	0.0009 (16)
C7	0.034 (2)	0.029 (2)	0.043 (2)	0.0028 (16)	0.0017 (17)	0.0007 (17)
C8	0.046 (2)	0.035 (2)	0.044 (2)	0.0067 (18)	0.0077 (19)	−0.0071 (19)
C9	0.031 (2)	0.034 (2)	0.037 (2)	0.0013 (16)	0.0057 (17)	−0.0069 (17)
C10	0.069 (3)	0.035 (2)	0.047 (3)	0.011 (2)	0.003 (2)	0.005 (2)
C11	0.057 (3)	0.059 (3)	0.034 (2)	0.010 (2)	0.011 (2)	−0.008 (2)

### Geometric parameters (Å, °)

**Table:**

Cu1—N1	1.963 (3)	C2—H2	0.9300
Cu1—O2	1.964 (3)	C3—C4	1.497 (6)
Cu1—O1	2.085 (3)	C4—H4A	0.9600
Cu1—N4	2.094 (3)	C4—H4B	0.9600
Cu1—O4^i^	2.125 (3)	C4—H4C	0.9600
Cu1—S1	2.5939 (11)	C5—H5A	0.9600
S1—O3	1.439 (3)	C5—H5B	0.9600
S1—O4	1.470 (3)	C5—H5C	0.9600
S1—O1	1.479 (3)	C6—H6A	0.9700
S1—O2	1.513 (3)	C6—H6B	0.9700
O4—Cu1^i^	2.125 (3)	C7—C8	1.365 (5)
N1—C1	1.333 (5)	C7—C10	1.489 (6)
N1—N2	1.372 (4)	C8—C9	1.390 (6)
N2—C3	1.348 (5)	C8—H8	0.9300
N2—C6	1.444 (5)	C9—C11	1.499 (5)
N3—C7	1.354 (5)	C10—H10A	0.9600
N3—N4	1.366 (4)	C10—H10B	0.9600
N3—C6	1.449 (5)	C10—H10C	0.9600
N4—C9	1.326 (5)	C11—H11A	0.9600
C1—C2	1.394 (5)	C11—H11B	0.9600
C1—C5	1.486 (6)	C11—H11C	0.9600
C2—C3	1.367 (6)		
			
N1—Cu1—O2	168.29 (12)	C3—C2—H2	126.4
N1—Cu1—O1	100.44 (11)	C1—C2—H2	126.4
O2—Cu1—O1	69.90 (11)	N2—C3—C2	106.4 (3)
N1—Cu1—N4	91.60 (12)	N2—C3—C4	122.8 (4)
O2—Cu1—N4	98.70 (12)	C2—C3—C4	130.8 (4)
O1—Cu1—N4	117.24 (12)	C3—C4—H4A	109.5
N1—Cu1—O4^i^	89.80 (12)	C3—C4—H4B	109.5
O2—Cu1—O4^i^	93.50 (11)	H4A—C4—H4B	109.5
O1—Cu1—O4^i^	139.06 (11)	C3—C4—H4C	109.5
N4—Cu1—O4^i^	101.80 (12)	H4A—C4—H4C	109.5
N1—Cu1—S1	134.03 (9)	H4B—C4—H4C	109.5
O2—Cu1—S1	35.47 (8)	C1—C5—H5A	109.5
O1—Cu1—S1	34.73 (8)	C1—C5—H5B	109.5
N4—Cu1—S1	115.22 (9)	H5A—C5—H5B	109.5
O4^i^—Cu1—S1	117.63 (8)	C1—C5—H5C	109.5
O3—S1—O4	111.17 (17)	H5A—C5—H5C	109.5
O3—S1—O1	112.93 (18)	H5B—C5—H5C	109.5
O4—S1—O1	110.82 (18)	N2—C6—N3	111.7 (3)
O3—S1—O2	111.43 (17)	N2—C6—H6A	109.3
O4—S1—O2	108.23 (17)	N3—C6—H6A	109.3
O1—S1—O2	101.81 (16)	N2—C6—H6B	109.3
O3—S1—Cu1	132.76 (13)	N3—C6—H6B	109.3
O4—S1—Cu1	115.83 (12)	H6A—C6—H6B	107.9
O1—S1—Cu1	53.44 (11)	N3—C7—C8	105.6 (3)
O2—S1—Cu1	48.89 (11)	N3—C7—C10	123.2 (4)
S1—O1—Cu1	91.83 (14)	C8—C7—C10	131.1 (4)
S1—O2—Cu1	95.64 (14)	C7—C8—C9	107.0 (4)
S1—O4—Cu1^i^	114.00 (17)	C7—C8—H8	126.5
C1—N1—N2	106.1 (3)	C9—C8—H8	126.5
C1—N1—Cu1	134.3 (3)	N4—C9—C8	110.4 (3)
N2—N1—Cu1	119.6 (2)	N4—C9—C11	121.7 (4)
C3—N2—N1	110.9 (3)	C8—C9—C11	127.9 (4)
C3—N2—C6	129.9 (3)	C7—C10—H10A	109.5
N1—N2—C6	118.6 (3)	C7—C10—H10B	109.5
C7—N3—N4	111.8 (3)	H10A—C10—H10B	109.5
C7—N3—C6	130.3 (3)	C7—C10—H10C	109.5
N4—N3—C6	117.8 (3)	H10A—C10—H10C	109.5
C9—N4—N3	105.1 (3)	H10B—C10—H10C	109.5
C9—N4—Cu1	136.3 (3)	C9—C11—H11A	109.5
N3—N4—Cu1	117.0 (2)	C9—C11—H11B	109.5
N1—C1—C2	109.3 (3)	H11A—C11—H11B	109.5
N1—C1—C5	121.1 (3)	C9—C11—H11C	109.5
C2—C1—C5	129.6 (4)	H11A—C11—H11C	109.5
C3—C2—C1	107.3 (3)	H11B—C11—H11C	109.5

Symmetry codes: (i) −*x*, −*y*+1, −*z*.

## References

[bb1] Arnold, P. J., Davies, S. C., Dilworth, J. R., Durrant, M. C., Griffiths, D. V., Hughes, D. L., Richards, R. L. & Sharpe, P. C. (2001). *J. Chem. Soc. Dalton Trans.* pp. 736–746.

[bb2] Dhar, S., Nethaji, M. & Chakravarty, A. R. (2004). *J. Chem. Soc. Dalton Trans.* pp. 4180–4184. 10.1039/b414639e 15573170

[bb3] Endres, H., Noethe, D., Rossato, E. & Hatfield, W. E. (1984). *Inorg. Chem.* **23**, 3467–3473.

[bb4] Hatzidimitriou, A. G., Kapnisti, M. & Voutsas, G. (2006). *Z. Kristallogr. New Cryst. Struct.* **221**, 532–534.

[bb5] He, Y.-K. & Han, Z.-B. (2006). *Acta Cryst.* E**62**, m2676–m2677.

[bb6] Jacobson, R. (1998). Private communication to the Rigaku Corporation.

[bb7] Rigaku/MSC, (2001). *CrystalClear* Rigaku/MSC, The Woodlands, Texas, USA.

[bb8] Rigaku/MSC, (2004). *CrystalStructure* Rigaku/MSC, The Woodlands, Texas, USA.

[bb9] Sheldrick, G. M. (2008). *Acta Cryst.* A**64**, 112–122. 10.1107/S0108767307043930 18156677

[bb10] Springsteen, C. H., Sweeder, R. D. & LaDuca, R. L. (2006). *Cryst. Growth Des.* **6**, 2308–2314.

[bb11] Tamasi, G. & Cini, R. (2003). *J. Chem. Soc. Dalton Trans.* pp. 2928–2936.

[bb12] Thompson, L. K., Xu, Z. Q., Goeta, A. E., Howard, J. A. K., Clase, H. J. & Miller, D. O. (1998). *Inorg. Chem.* **37**, 3217–3229. 10.1021/ic9715761 11670453

